# Sex differences and the roles of sex steroids in energy intake regulation and high-fat diet preference during free-choice feeding in young adult rats

**DOI:** 10.3389/fendo.2026.1864440

**Published:** 2026-06-18

**Authors:** Natsumi Kosugi, Aoi Takahashi, Nanako Sakagawa, Mio Nishimaki, Keiko Morimoto, Akira Takamata

**Affiliations:** 1 Department of Environmental Health, Nara Women’s University, Nara, Japan; 2 Department of Health and Nutrition, Kyoto Koka Women’s University, Kyoto, Japan

**Keywords:** androgens, estrogens, food preference, high-fat diet, nucleus accumbens, obesity

## Abstract

Excessive intake of high-fat diet (HFD) contributes to obesity. Sex differences and the specific roles of sex steroids in regulating HFD preference, particularly in metabolically flexible young animals, remain poorly understood. We first examined energy intake in young (starting age: 8 weeks old) gonadally intact male and female rats provided with *ad libitum* access to either a normal diet (ND) or an HFD alone for 30 days, without a dietary choice. Energy intake did not differ between ND-fed and HFD-fed rats in either sex. Therefore, we hypothesized that the lack of a dietary choice may mask the preference for HFD. In the next experiment, young rats were assigned to six groups: gonadally intact males (M) and females (F), sham-operated (Sham) or orchiectomized (ORX) males, and ovariectomized females with or without estradiol replacement (E2(+), E2(–)). Animals received either ND alone or concurrent access to ND and HFD for 30 days. HFD preference was significantly greater in the M than in the F group. HFD preference and total energy intake were greater in the M and Sham groups than in the ORX group when provided with HFD. In contrast, HFD preference did not differ among the F, E2(–), and E2(+) groups, although E2 deficiency increased total energy intake regardless of diet condition. Chronic neuronal activity was assessed via ΔFosB expression in the nucleus accumbens (NAc). ΔFosB expression in the NAc was significantly higher in the HFD-fed than in the ND-fed rats in the M group. These results suggest that androgens play a critical role in enhancing HFD preference and excessive energy intake under free–choice conditions in young adult male rats. In contrast, under the present experimental conditions, estrogens primarily regulate total energy intake but do not affect HFD preference, suggesting mechanistically distinct pathways for dietary choice between sexes.

## 1 Introduction

Obesity has become a major global health concern because it increases the risks of metabolic and cardiovascular diseases, including diabetes mellitus, hyperlipidemia, hypertension, and other cardiovascular disorders (1–4). Obesity primarily results from an imbalance between energy intake and energy expenditure, most commonly due to excessive energy consumption. In modern societies, highly palatable, inexpensive, and energy-dense foods are readily accessible, increasing the prevalence of excessive energy intake (5, 6).

Feeding behavior is regulated by two systems: a homeostatic system that maintains the energy balance and a hedonic system that drives the motivation and pleasure associated with consuming palatable foods (7, 8). In mammals, including humans, feeding behavior exhibits sex differences that emerge after the onset of puberty, with males generally consuming more food than females (9). These differences are thought to be largely mediated by the effects of sex steroid hormones on feeding-related neural systems (9). Additionally, sex differences have been documented in taste preferences (10–14) and in susceptibility to overeating (15, 16).

Sex steroid hormones are likely to influence both homeostatic and hedonic systems. In the homeostatic system, estrogens robustly exert anorexigenic and anti-obesity effects (9, 17, 18). This finding is further supported by epidemiological observations showing that the prevalence of obesity increases after menopause as circulating estrogen levels decline (19), as well as by animal studies demonstrating that estrogen replacement in ovariectomized rats reduces food intake and weight gain (9). In contrast, the effects of androgens on feeding regulation remain controversial, with both orexigenic and anorexigenic effects reported (9, 20–23).

Regarding the hedonic system, estradiol replacement in ovariectomized rats increases the intake of palatable sweetened solutions while reducing normal diet intake, suggesting that estradiol not only suppresses overall energy intake but also promotes the consumption of palatable sweet fluids (24). In contrast, the role of androgens in hedonic feeding remains largely unexplored. Notably, androgen deficiency has been reported to exacerbate high-fat diet (HFD)-induced metabolic disturbances, including increased adiposity, impaired glucose homeostasis (25), and hepatic steatosis (26). These metabolic alterations are often associated with changes in food preference, suggesting that sex steroid hormones may coordinately regulate both metabolic and hedonic pathways to shape sex-specific feeding patterns.

Although HFD feeding is widely used as an experimental model of obesity, and sex differences in feeding behavior under HFD conditions have been well documented (27–31), the specific role of sex steroid hormones in these responses remains poorly understood, particularly during young adulthood (8–12 weeks of age in rats). During this stage, immediately following the completion of gonadal maturation, the neural circuits governing both homeostatic and hedonic feeding are fully functional, yet the metabolic defense against HFD-induced obesity may be more robust than in older adults (32–34). However, the specific influence of sex steroids on the preference for high-fat stimuli during this period remains less clear.

In the present study, we first measured food (energy) consumption in gonadally intact young adult (starting age: 8 weeks old) male and female rats given *ad libitum* access to either an HFD alone or a normal diet (ND) without a dietary choice for 30 days (Experiment 1). Unexpectedly, energy intake did not differ between HFD-fed and ND-fed rats of either sex. Therefore, we hypothesized that exclusive access to an HFD may attenuate the preference for the HFD.

We next examined the feeding behaviors of gonadally intact young adult male and female rats using a free-choice paradigm, in which rats were provided *ad libitum* access to both an HFD and an ND simultaneously or an ND alone for 30 days (Experiment 2) to test this hypothesis. We further assessed the roles of sex steroids by applying the same paradigm to ovariectomized female rats with or without chronic estradiol replacement and to orchiectomized and sham-operated male rats. On the final day of the experiment, brain samples were collected to evaluate the long-term neuronal activation in the nucleus accumbens (NAc) by assessing ΔFosB expression to explore the underlying mechanisms (35, 36).

## 2 Materials and methods

### 2.1 Ethical approval and animals

All experiments were conducted in accordance with the “Standards Relating to the Care and Keeping and Reducing Pain of Laboratory Animals” established by the Ministry of Environment of Japan and the Guiding Principles for the Care and Use of Animals in the Field of Physiological Sciences, as outlined by the Physiological Society of Japan. All experimental procedures were approved by the Ethics Committee for Animal Care and Use of Nara Women’s University (approval #22-02).

Male and female Wistar rats (Jcl: Wistar; CLEA Japan, Inc., Japan), 6 to 7 weeks old upon arrival, were used in all the experiments. Initial body weights, at 8 weeks of age, immediately before the onset of the HFD-feeding period, are shown in **Table 1** and **2**. For the first experiment (Experiment 1), the rats were housed individually in plastic cages (258 × 258 × 356 mm) equipped with a feeding monitoring apparatus (MODEL FIC-001; Muromachi Kikai Co., Ltd., Japan). For the second experiment (Experiment 2), the rats were housed individually in plastic cages (250 × 410 × 190 mm) with a stainless-steel breeding lid top. All the cages were placed in a chamber maintained on a 12/12-h light/dark cycle (Experiment 1: lights on at 0500 h and off at 1700 h; Experiment 2: lights on at 0700 h and off at 1900 h), with an ambient temperature of 22–23 °C and a relative humidity of 45% throughout the experimental periods.

**Table 1:** Body weight before the experiment in male and female rats (experiment 1).

Group	Diet	Initial B.W. (g)	
		Mean	± SEM
M	ND	264.1^a^	4.2
	HFD	263.4^a^	4.4
F	ND	184.5^b^	1.9
	HFD	183.0^b^	1.8

Intact male (M) and female (F) rats were assigned to a normal diet (ND) fed group or a high-fat diet (HFD) fed group. Body weight was measured daily at ZT11 (1600 h). Data are presented as means ± SEMs (n = 6 rats per group). Different small letters indicate significant differences among groups analyzed using Tukey’s Honest Significant Difference.

**Table 2:** Body weight before the experiment (day 0) and on the day 10 in male and female rats (experiment 2).

Group	Diet	Initial B.W. (g)		Day 10 B.W. (g)	
		Mean	± SEM	Mean	± SEM
M	ND	277.3^a^	3.4	325.6^a^	4.3
	HFD	276.6^a^	2.6	334.7^a^	4.8
F	ND	191.8^b^	2.8	217.6^b^	4.2
	HFD	191.7^b^	2.4	222.1^b^	2.4
M	ND	277.3^a^	3.4	325.6^a^	4.3
	HFD	276.6^a^	2.6	334.7^a^	4.8
Sham	ND	268.9^a^	3.1	319.8^abc^	2.9
	HFD	268.2^a^	4.7	324.2^ab^	5.9
ORX	ND	250.8^b^	5.0	300.7^c^	5.4
	HFD	250.5^b^	3.7	304.0^bc^	5.3
F	ND	191.8^a^	2.8	217.6^c^	4.2
	HFD	191.7^a^	2.4	222.1^bc^	2.4
E2(–)	ND	187.0^a^	3.7	235.6^ab^	5.7
	HFD	187.0^a^	2.9	241.0^a^	4.5
E2(+)	ND	172.6^b^	1.4	199.3^d^	1.8
	HFD	173.0^b^	2.0	200.8^d^	2.0

Rats were assigned to six groups: gonadally intact males (M), gonadally intact females (F), sham-operated males (Sham), orchiectomized males (ORX), ovariectomized females without estradiol replacement [E2(–)], and ovariectomized females with estradiol replacement [E2(+)]. Rats were given either access to a normal diet (ND) alone or simultaneous access to a normal diet and a high-fat diet (HFD) for 30 days. Body weight was measured daily at ZT3 (1000 h). Data are presented as means ± SEMs. Comparisons were made between the M and F groups (upper), among the M, Sham, and ORX groups (middle), and among the F, E2(–), and E2(+) groups (lower) (n = 9 rats in the F-ND and F-HFD groups; n = 8 rats in the other groups). Different small letters indicate significant differences among groups analyzed using Tukey’s Honest Significant Difference.

All the rats had *ad libitum* access to a normal rodent diet (ND, CE-2; CLEA Japan, Inc., Japan: 3.424 kcal/g, containing 12.5% fat, 29.2% protein, and 58.2% carbohydrates by caloric content) and/or a high-fat diet (HFD; HFD-60; Oriental Yeast Co., Ltd., Japan: 5.062 kcal/g, containing 62.2% fat, 18.2% protein, and 19.6% carbohydrates by caloric content), along with tap water. The feeding conditions varied among the experiments and the groups within each experiment (see below), and the feeding experiments were initiated when the rats were 8 weeks of age in all the experiments. The HFD was replaced daily with fresh food to prevent flavor deterioration due to lipid autooxidation.

### 2.2 Experimental design

#### 2.2.1 HFD feeding with no dietary choice (experiment 1)

In Experiment 1, we examined sex differences in HFD intake behavior with no dietary choice. Gonadally intact male (n = 12; M group) and female (n = 12; F group) rats were subdivided into two groups: the HFD group (M-HFD, n = 6; F-HFD, n = 6) and the ND group (M-ND, n = 6; F-ND, n = 6). Their body weight was measured daily at ZT11 (1600 h), and food intake was monitored for 30 days using a feeding monitoring apparatus.

#### 2.2.2 HFD feeding with a dietary choice (experiment 2)

In Experiment 2, feeding experiments were conducted under a dietary choice condition. To examine sex differences, gonadally intact male (n = 16; M group) and female (n = 18; F group) rats were assigned to either the ND or the HFD group; male rats were divided into the M-ND (n = 8) and M-HFD (n = 8) groups, and female rats were divided into the F-ND (n = 9) and F-HFD (n = 9) groups. To examine the role of androgens in male rats, 7-week-old male rats (n = 32) were assigned to either the orchiectomy (ORX) group (n = 16) or the sham-operated (Sham) group (n = 16). Following a 7-day recovery period after surgery, the ORX and Sham groups were subdivided into the ND and HFD groups: the ORX-ND, ORX-HFD, Sham-ND, and Sham-HFD groups (n = 8 rats per group). To examine the role of estrogens in females, 7-week-old female rats (n = 32) were ovariectomized and divided into two groups: an estradiol-treated (E2(+)) group and a cholesterol-treated (estrogen-deficit; E2(–)) group. Following a 7-day recovery period after ovariectomy and hormone replacement, the E2(+) and E2(–) groups were subdivided into the ND and HFD groups: the E2(+)-ND, E2(+)-HFD, E2(–)-ND, and E2(–)-HFD groups (n = 8 rats per group). Details of the feeding protocol are described in 2.4.

### 2.3 Surgery

#### 2.3.1 Orchiectomy and sham operation

Bilateral orchiectomy was performed using a dorsal approach under general anesthesia induced by an intraperitoneal injection of a cocktail of butorphanol tartrate (3.75 mg/kg), midazolam (3.0 mg/kg), and medetomidine hydrochloride (0.225 mg/kg) in saline. Additional doses were administered during the surgery if necessary. In sham-operated rats, the testes were exposed and gently touched with forceps but not removed. After surgery, the rats were administered gentamicin sulfate to prevent infection from the incisions.

#### 2.3.2 Ovariectomy and hormone replacement

Bilateral ovariectomy was performed via a ventral approach under the same general anesthesia described above. After ovariectomy, a silicon capsule containing either 17β-estradiol or cholesterol was implanted subcutaneously through an interscapular incision. The capsule was prepared from silicon tubing (25 mm long, 2 mm inner diameter, 3 mm outer diameter) filled with either cholesterol alone for the E2(–) group or a mixture of 17β-estradiol and cholesterol (1:4 by weight) for the E2(+) group. Both ends of the tubing were sealed with silicon adhesive. The steroid or cholesterol was released via diffusion through the silicone tubing, providing sustained proestrus estradiol levels over the experiments (17, 37). The capsules were immersed in physiological saline for at least one day before implantation. After surgery, the rats were administered gentamicin sulfate to prevent infection.

### 2.4 Feeding protocol, measurement, and calculation

In Experiment 2, the HFD and ND were placed separately on a stainless-steel breeding lid top with a divider, and their left and right positions were switched daily in the HFD group. In the ND group, the ND was placed in both compartments. The rats were allowed free access to these diets and bottled tap water. The feeding trial lasted for 30 days, during which time body weight and diet intake were measured daily at ZT3 (1000 h). Daily food and water intake were calculated as the difference between the weight recorded 24 h earlier and the weight remaining after 24 h. In addition, we calculated the percentage of energy intake from the HFD relative to total energy intake as an index of HFD preference in the HFD-fed groups.

### 2.5 Tissue collection

After completing the feeding trials, the rats were deeply anesthetized with sodium pentobarbital (> 65 mg/kg, i.p.). Blood samples were collected by cardiac puncture. The rats were then perfused transcardially with ice-cold phosphate-buffered saline containing heparin (2.0 U/mL). The brains were subsequently fixed by transcardial perfusion with 4% paraformaldehyde in 0.1 M sodium phosphate buffer (PB) and removed.

Blood samples were centrifuged, and the collected serum samples were stored at –50 °C until analysis. The brains were postfixed with the same fixative for 1–2 days at 4 °C and immersed in 15% (w/v) sucrose in PBS for 1 day and then in 25% sucrose in PBS for 2 days for cryoprotection. Finally, frozen brains were coronally sectioned at 30 µm using a cryostat microtome for a subsequent immunohistochemical analysis of ΔFosB expression in the nucleus accumbens (NAc).

### 2.6 Immunohistochemical analysis

We performed an immunohistochemical examination of ΔFosB expression to examine long-term neuronal activation in the NAc. Free-floating sections were blocked with 3% normal goat serum, incubated overnight at 4 °C with an anti-ΔFosB antibody (1:20, 000 dilution; Delta FosB (D3S8R) rabbit mAb #14695, Cell Signaling Technology, Danvers, MA, USA), and incubated with a biotinylated secondary antibody (1:400 dilution; BA-1000, Vector, Burlingame, CA, USA) for 2 h, followed by an incubation with the ABC Elite kit solution (1:400 dilution; PK-6100, Vector, Burlingame, CA, USA) for 2 h. The staining was visualized with 0.02% 3, 3’-diaminobenzidine (DAB) and 0.01% H_2_O_2_ in 50 mM Tris HCl buffer (pH 7.4). The sections were mounted on gelatin-coated glass slides, dehydrated with graded ethanol solutions, cleared with Lemosol^®^, and cover slipped with mounting medium (Mount Quick; Daido Sangyo Co., Ltd., Japan).

From the series of coronal sections containing the NAc identified using a rat brain atlas (38), three sections were selected at intervals of every fifth section for observation. Bright-field images of ΔFosB staining were captured (BZ-X710; Keyence Co., Japan). The density of ΔFosB-immunoreactive (ΔFosB-ir) cells in the NAc core and shell was analyzed bilaterally using ImageJ software (version 1.53; National Institutes of Health, USA). and the mean value of both sides—or the value of the single intact side when the other was damaged in a few rats—was used for statistical analysis. The mean density (cells/mm^2^) across 3 sections for each animal was used for the statistical analysis. The analysis was performed by an investigator blinded to the experimental treatment.

### 2.7 Serum sex steroid analyses

In Experiment 2, serum testosterone concentrations in the Sham and ORX groups were determined using a competitive enzyme-linked immunosorbent assay (ELISA) (Testosterone ELISA Kit; Cayman Chemical Co., USA). Serum estradiol concentrations in the F, E2(+), and E2(–) groups were determined using a competitive ELISA (Estradiol ELISA Kit; Cayman Chemical Co., USA).

### 2.8 Statistical analyses

All data are presented as means ± SEMs. Statistical analyses were performed using jamovi (version 2.6.45.0). In Experiment 2, data from Days 11–30 following the 10-day acclimation period were used for group comparisons. Serum testosterone and estradiol concentrations were log-transformed prior to analysis to address heterogeneity of variance between hormonal conditions. Statistical significance was evaluated using one- or two-way ANOVA followed by Tukey’s *post hoc* test, or an unpaired Student’s t-test, as appropriate. Correlations were analyzed using linear regression model. A P value < 0.05 was considered statistically significant. P values for main effects and interactions from the one- or two-way ANOVA and unpaired Student’s t-test are shown in **Supplementary Table 1**.

## 3 Results

### 3.1 Experiment 1

**Figure 1:**
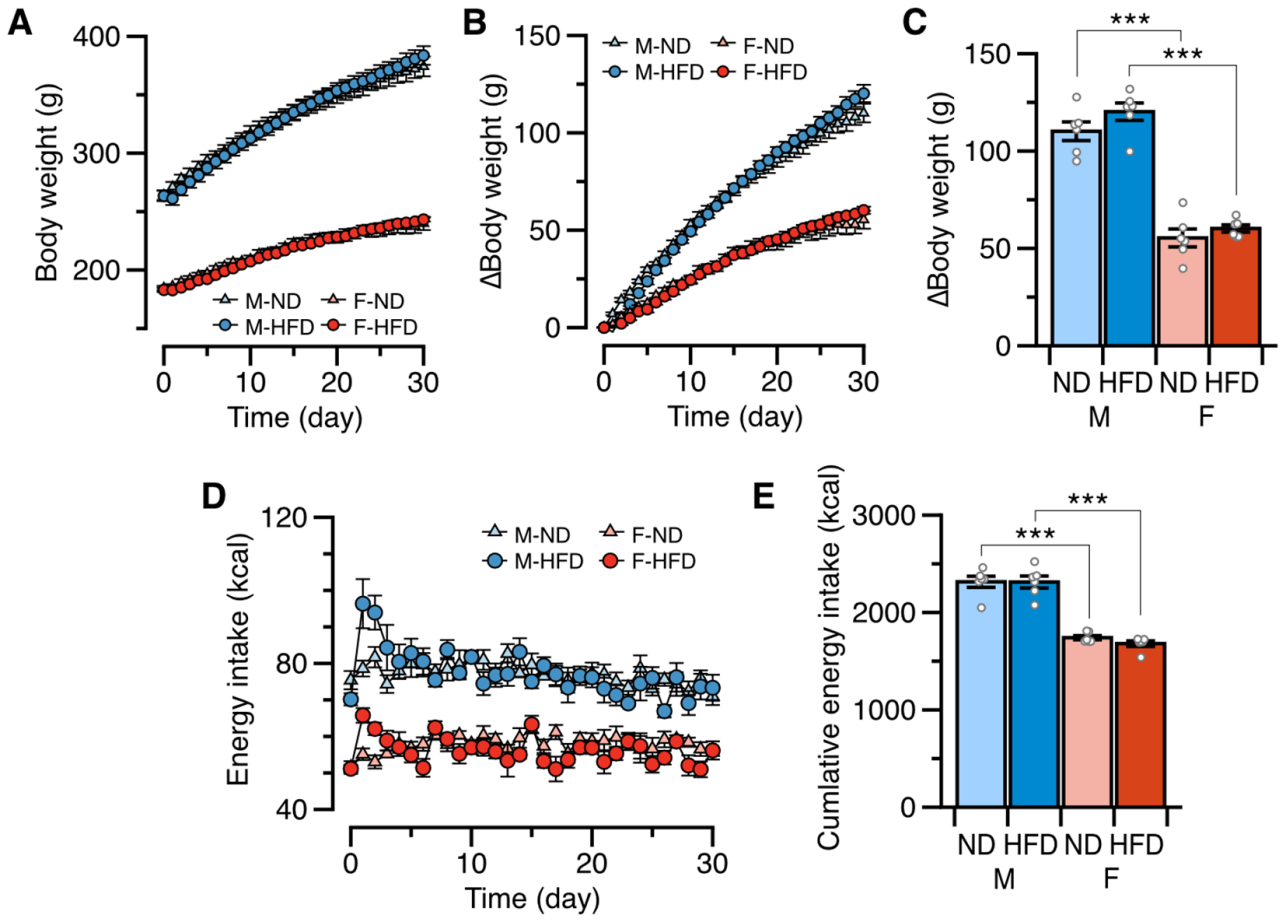
Body weight, body weight gain, and energy intake during the 30-day feeding period in male and female rats (Experiment 1). Gonadally intact male (M) and female (F) rats were allowed *ad libitum* access to a normal diet (ND) or a high-fat diet (HFD), without dietary choice, along with water for 30 days. Panels show the time course of absolute body weight **(A)**, body weight gain **(B)**, body weight gain on the final experimental day **(C)**, the time course of energy intake **(D)**, and cumulative energy intake **(E)**. Data are presented as means ± SEMs (n = 6 rats per group). *** indicates a significant difference between male and female rats in the corresponding diet group (p < 0.001).

Initial body weight was significantly greater in the M than in the F group regardless of diet conditions (ND or HFD). Importantly, there was no significant difference in initial body weight between the ND and HFD groups in either sex (**Table 1**). Body weight gain and cumulative energy intake over the 30-day feeding period were significantly greater in the M group than in the F group under both ND and HFD conditions (**Figures 1A–E**). However, HFD feeding did not significantly affect either energy intake or body weight gain in rats of either sex (**Figures 1A–E**).

### 3.2 Experiment 2

#### 3.2.1 Serum sex steroid concentrations

**Figure 2:**
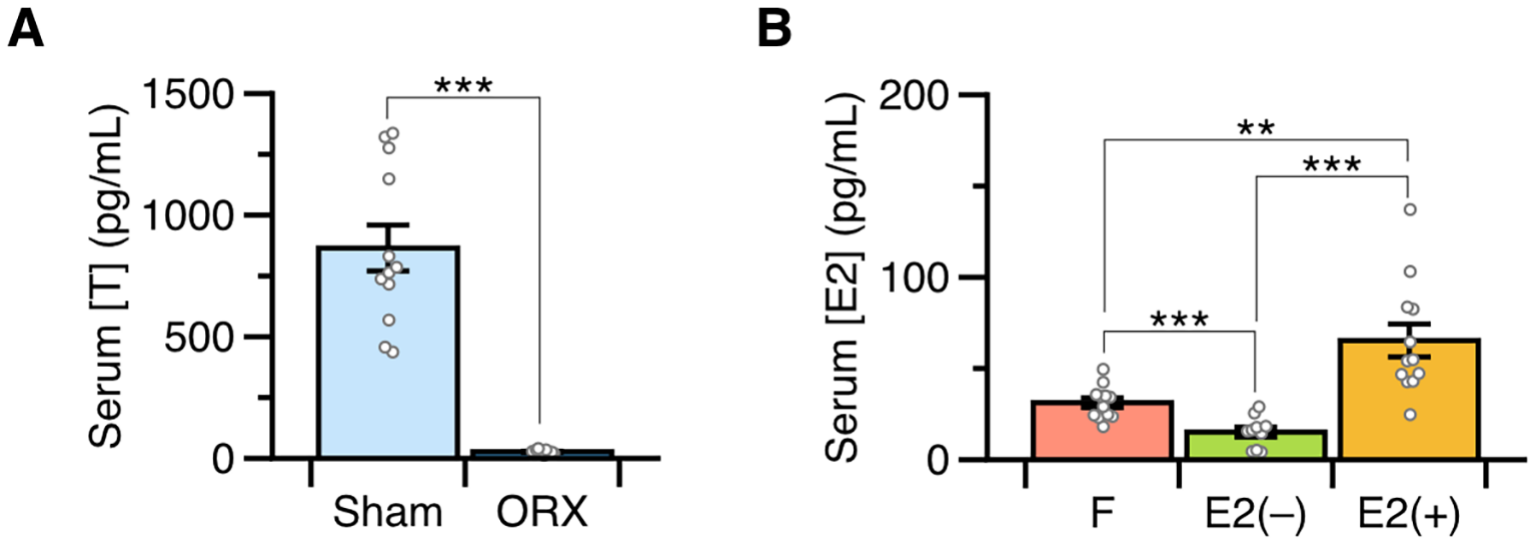
Serum testosterone **(A)** and estradiol **(B)** concentrations in male and female rats after the 30-day feeding period (Experiment 2). Sham, sham-operated male; ORX, orchiectomized male; F, gonadally-intact female; E2(–), ovariectomized females without estradiol replacement; and E2(+), ovariectomized females with estradiol replacement. Testosterone and estradiol concentrations were log-transformed prior to statistical analysis to address heterogeneity of variance among the conditions. Data are presented as means ± SEMs (n = 10 rats in the E2(–) group; n = 12 rats in the other groups). ** and *** indicate significant differences between groups at p < 0.01 and p < 0.001, respectively.

Data from the ND and HFD groups were pooled within each gonadal status regardless of diet because feeding conditions did not affect serum sex steroid concentrations (**Figures 2A, B**). Serum testosterone concentrations were lower in the ORX group than in the Sham group (**Figure 2A**). Serum estradiol concentrations were highest in the E2(+) group, intermediate in the F group, and lowest in the E2(–) group (**Figure 2B**).

#### 3.2.2 Body weight

**Figure 3:**
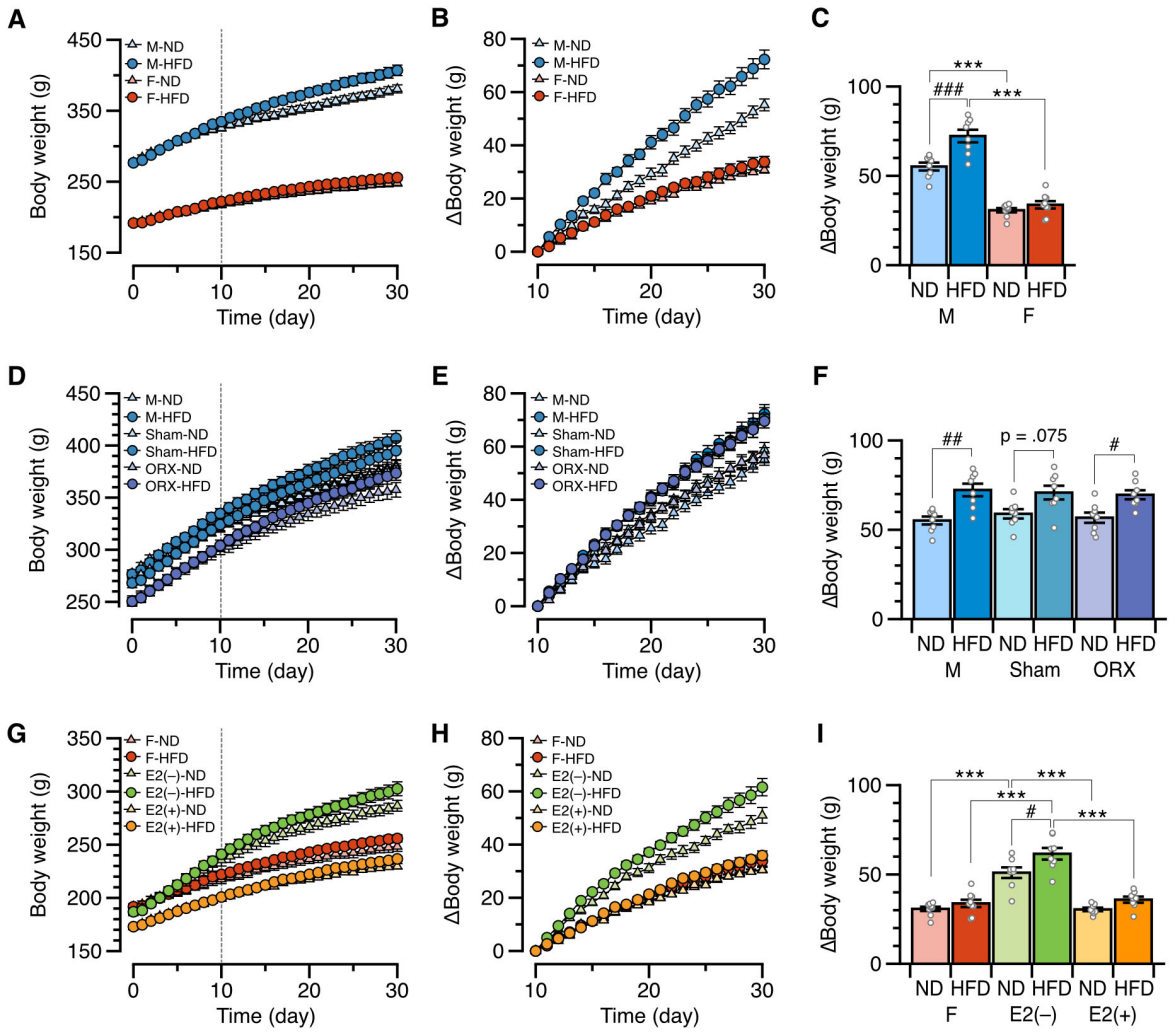
Body weight and body weight gain from Day 10 to Day 30 in male and female rats under different hormonal statuses (Experiment 2). Rats were assigned to six groups: gonadally intact males (M), gonadally intact females (F), sham-operated males (Sham), orchiectomized males (ORX), ovariectomized females without estradiol replacement (E2(–)), and ovariectomized females with estradiol replacement (E2(+)). Rats were given either access to a normal diet (ND) alone or simultaneous access to a normal diet and a high-fat diet (HFD), with dietary choice, along with water. Panels show the time course of absolute body weight during the 30-day feeding period **(A, D, G)**, the time course of body weight gain from Day 10 to Day 30 **(B, E, H)**, and the body weight gain on the final experimental day from Day 10 **(C, F, I)** for intact male and female **(A–C)**, male groups **(D–F)** and female groups **(G–I)**. Data are presented as means ± SEMs (n = 9 rats in the F-ND and F-HFD groups; n = 8 rats in the other groups). *** indicates significant differences within hormonal status at p < 0.001. #, ##, and ### indicate significant differences between diets at p < 0.05, p < 0.01, and p < 0.001, respectively.

Time course of body weight during the 30-day feeding period is shown in **Figures 3A, D, G**. Body weight at Day 0 and Day 10 are summarized in **Table 2**. Initial body weight before the HFD presentation (at Day 0) and at Day 10 were significantly greater in the M than in the F group. Among males, the initial body weight at Day 0 was significantly smaller in the ORX than in the M and Sham groups, and the body weight at Day 10 remained significantly lower in the ORX than in the M group. Among females, the body weight at Day 0 was significantly smaller in the E2(+) than in the F and E2(–) groups. By Day 10, the body weight was highest in the E2(–) group, intermediate in the F group, and lowest in the E2(+) group. Importantly, feeding condition (ND or HFD) did not affect body weight on either Day 0 or Day 10 in any of the groups (**Table 2**).

Body weight gain over the 20-day feeding period (Days 10–30) was significantly greater in the M-HFD group than in the M-ND group, whereas HFD feeding had no effect on body weight gain in the F group (**Figures 3B, C**). Among the male groups (M, Sham, and ORX), body weight gain was significantly greater in the HFD group than in the ND group in both the M and ORX groups, and tended to be greater in the Sham-HFD group than in the Sham-ND group (p = 0.075) (**Figures 3E, F**). Hormonal conditions did not affect body weight changes in males (**Figures 3E, F**). In contrast, among the female groups (F, E2(–), and E2(+)), body weight gain was significantly greater in the E2(–) groups than in the F and E2(+) groups regardless of diet conditions (**Figures 3H, I**). HFD feeding significantly increased body weight gain in the E2(–) groups, while having no significant impact in the F or E2(+) groups (**Figures 3H, I**).

#### 3.2.3 Feeding behavior

##### 3.2.3.1 Cumulative total energy intake

**Figure 4:**
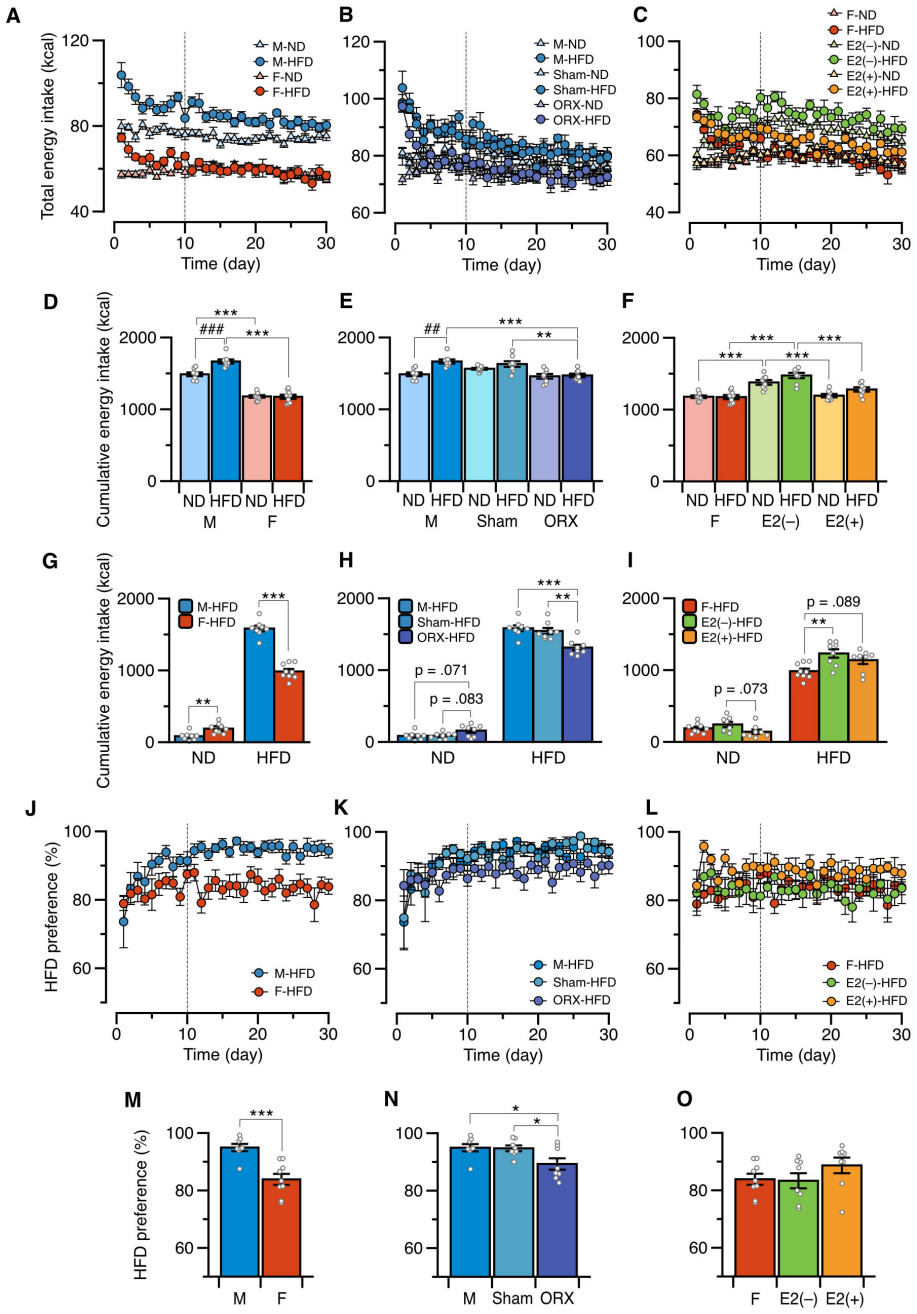
Cumulative total energy intake and HFD preference in male and female rats under different hormonal statuses (Experiment 2). Rats were assigned to six groups: gonadally intact males (M), gonadally intact females (F), sham-operated males (Sham), orchiectomized males (ORX), ovariectomized females without estradiol replacement (E2(–)), and ovariectomized females with estradiol replacement (E2(+)). Rats were given either access to a normal diet (ND) alone or simultaneous access to a normal diet and a high-fat diet (HFD), with dietary choice, along with water for 30 days. Panels show the time courses of daily total energy intake over 30 days **(A, B, C)**, cumulative total energy intake during the 20-day feeding period **(D, E, F)**. For the HFD-fed groups, panels show cumulative energy intake from ND and HFD during the 20-day feeding period **(G, H, I)**, and time course of HFD preference **(J, K, L)**, and mean HFD preference from Day 11 to Day 30 **(M, N, O)**. Comparisons were made between the M and F **(A, D, G, J, M)**, among the M, Sham, and ORX **(B, E, H, K, N)**, and among the F, E2(–), and E2(+) groups **(C, F, I, L, O)**. HFD preference was defined as the percentage of energy intake from the HFD relative to total energy intake. Food intake was measured daily at ZT3 (1000 h). Data are presented as means ± SEMs (n = 9 rats in the F-ND and F-HFD groups; n = 8 rats in the other groups). *, **, and *** indicate significant differences within hormonal status at p < 0.05, p < 0.01, and p < 0.001, respectively. ## and ### indicate significant differences between diets at p < 0.01 and p < 0.001, respectively.

Time course of total energy intake during the 30-day feeding period is shown in **Figures 4A-C**. Cumulative total energy intake over the 20-day period was higher in the M group than in the F group in both the HFD and ND groups (**Figure 4D**). In addition, cumulative total energy intake over the 20-day period was significantly higher in the M-HFD group than in the M-ND group (**Figure 4D**). Among the HFD fed-males, cumulative total energy intake was significantly lower in the ORX group than in the M and Sham groups (**Figure 4E**). HFD increased cumulative total energy intake in the M group (**Figure 4E**). Among the female groups, cumulative total energy intake was significantly higher in the E2(–) group than in the F and E2(+) groups, regardless of diet conditions (**Figure 4F**). Dietary choice did not affect the energy intake in any of the female groups (**Figure 4F**).

##### 3.2.3.2 Cumulative energy intake derived from each diet (ND or HFD) in the HFD-fed rats

Cumulative energy intake from the ND over the 20-day period was significantly higher in the F-HFD group than in the M-HFD group, whereas cumulative energy intake from the HFD was significantly higher in the M-HFD group than in the F-HFD group (**Figure 4G**). Among the male groups, cumulative energy intake from ND tended to be greater in the ORX-HFD group than in the M-HFD group (p = 0.071) and Sham-HFD group (p = 0.083) (**Figure 4H**). Cumulative energy intake from HFD was significantly smaller in the ORX group than in the M and Sham groups (**Figure 4H**). Among the female groups, cumulative energy intake from ND tended to be higher in the E2(–) group than in the E2(+) group (p = 0.073) (**Figure 4I**). Cumulative energy intake from HFD was significantly higher in the E2(–) group than in the F group, and tended to be higher in the E2(+) group than in the F group (p = 0.089) (**Figure 4I**).

##### 3.2.3.3 HFD preference (percentage of energy intake from HFD)

Preference for HFD was calculated as the percentage of energy intake from the HFD relative to total energy intake. Time course of HFD preference during the 30-day feeding period is shown in **Figures 4J–L**. HFD preference was significantly greater in the M-HFD group than in the F-HFD group (**Figure 4M**). Among the male groups, HFD preference was significantly lower in the ORX group than in the M and Sham groups (**Figure 4N**), whereas there was no significant difference in HFD preference among the female groups (**Figure 4O**).

#### 3.2.4 ΔFosB expression in the NAc core and shell

**Figure 5:**
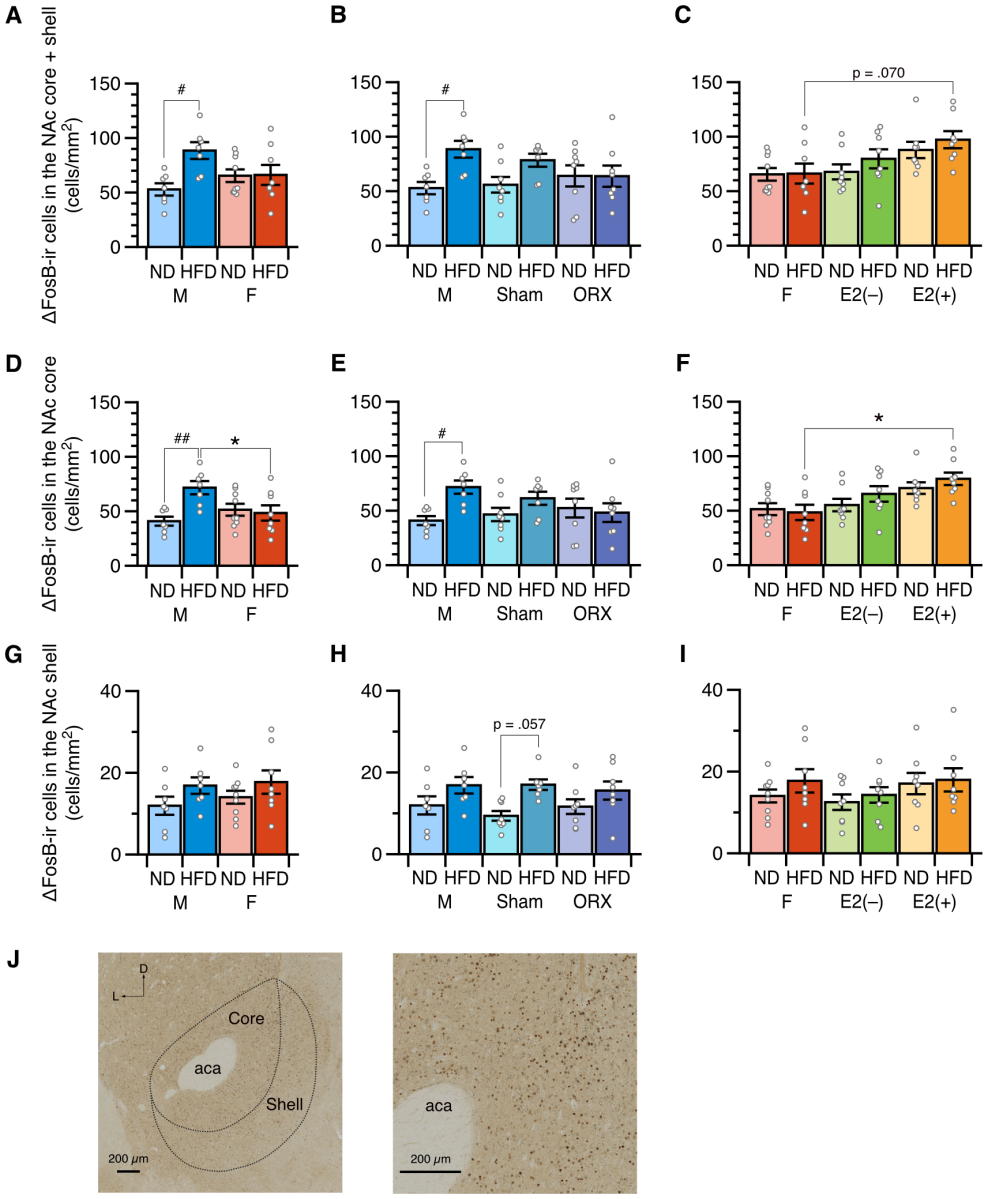
Density of ΔFosB-immunoreactive cells in the nucleus accumbens (Experiment 2). Rats were assigned to six groups: gonadally intact males (M), gonadally intact females (F), sham-operated males (Sham), orchiectomized males (ORX), ovariectomized females without estradiol replacement [E2(–)], and ovariectomized females with estradiol replacement [E2(+)]. Rats were given either access to a normal diet (ND) alone or simultaneous access to a normal diet and a high-fat diet (HFD), with dietary choice, along with water for 30 days. Brain samples were collected following the 30-day feeding period. Panels show the density of ΔFosB-immunoreactive (ΔFosB-ir) cells in the nucleus accumbens (NAc) core + shell **(A–C)**, core **(D–F)**, and shell **(G–I)**. Comparisons were made between the M and F **(A, D, G)**, among the M, Sham, and ORX **(B, E, H)**, and among the F, E2(–), and E2(+) groups **(C, F, I)**. A representative photomicrograph of ΔFosB-ir cells in the NAc is shown in **(J)** (scale bar = 200 µm). Data are presented as means ± SEMs (n = 7 rats in the M-ND, M-HFD, and Sham-HFD groups; n = 9 rats in the F-ND group; n = 8 rats in the other groups). * indicates significant difference within hormonal status at p < 0.05. # and ## indicate significant differences between diets at p < 0.05 and p < 0.01, respectively.

Representative images of ΔFosB expression in the NAc are shown in **Figure 5J** and S1. Two-way ANOVA followed by Tukey’s *post hoc* test revealed that ΔFosB expression in the NAc core + shell was significantly higher in the M-HFD group than in the M-ND group (**Figure 5A**). In addition, ΔFosB expression in the NAc core was significantly greater in the M-HFD group than in the M-ND and F-HFD groups (**Figure 5D**). Among the male groups, ΔFosB expression was significantly higher in the M-HFD group than in the M-ND group in the NAc core + shell and core (**Figures 5B, E**) and tended to be higher in the Sham-HFD group than in the Sham-ND group in the NAc shell (p = 0.057) (**Figure 5H**). No significant differences were observed between the ORX groups in ΔFosB expression (**Figures 5B, E, H**). Among the female groups, ΔFosB expression in the NAc core was significantly higher in the E2(+)-HFD group than in the F-HFD group (**Figure 5F**). Additionally, ΔFosB expression in the NAc core + shell tended to be higher in the E2(+)-HFD group than in the F-HFD group (p = 0.070) (**Figure 5C**). However, HFD feeding did not affect ΔFosB expression in any female group (**Figures 5C, F, I**).

ΔFosB expression in the NAc core + shell was positively correlated with cumulative total energy intake in males, but not in females (**Supplementary Figures 2B, C**), and the interaction between sex and cumulative total energy intake tended to be significant (p = 0.055), indicating that the association between energy intake and ΔFosB expression differed by sex (**Supplementary Figure 2A**). Furthermore, ΔFosB expression in the NAc core + shell did not significantly correlate with cumulative HFD intake or HFD preference in either males or females (**Supplementary Figures 2D–I**).

## 4 Discussion

The results of the present study demonstrate that free-choice access to HFD induces sex differences in feeding behavior and body weight regulation in young adult rats, whereas these differences are not observed when HFD is provided alone. Moreover, distinct sex steroid hormones contribute to these differences: androgens enhance HFD intake and preference, whereas estrogens primarily regulate energy intake and body weight gain in young adult rats.

HFD feeding is commonly used as an experimental model to induce obesity and metabolic disorders (27). In the present study, we found that 30-day HFD feeding without dietary choice (Experiment 1) did not result in excessive body weight gain or energy intake in young adult rats (8–12 weeks old) of either sex. These findings suggest that young adult rats exhibit high metabolic flexibility and relative resistance against diet-induced obesity compared to older adults (30, 32–34). Consistent with this, previous studies have shown that protective effects against HFD observed in young female mice diminish with age, leading to greater weight gain and impaired glucose tolerance (30).

However, the free-choice paradigm in the present study (Experiment 2) revealed that the availability of a palatable HFD alongside less palatable ND can override this metabolic resilience, particularly in the presence of androgens. Access to highly palatable foods with dietary choice likely stimulate sensory-specific and hedonic factors that override the innate metabolic buffering capacity of young rats, leading to sex-specific dietary selection (39). In the present study, free-choice access to HFD induced greater susceptibility in males. Notably, even under free-choice conditions, the increases in total energy intake and body weight gain remained relatively modest (29).

Gonadally intact male rats exhibited a stronger preference for HFD than females (**Figure 4M**). This preference was significantly attenuated by orchiectomy (**Figure 4N**). Consistently, in HFD-fed males, HFD intake was higher in androgen-intact males than orchiectomized males, whereas ND intake in these rats was unaffected by hormonal status (**Figure 4H**). In addition, hormonal status did not affect total energy intake in the ND-fed males (**Figure 4E**). These findings suggest that endogenous androgens selectively enhance hedonic feeding toward palatable, energy-dense HFD, rather than increasing overall energy intake per se. This interpretation is supported by previous studies showing that orchiectomy reduces food intake, whereas androgen replacement restores feeding behavior (9, 21, 40). In addition, chronic testosterone administration increases HFD preference in gonadally intact female rats, but not in ovariectomized rats (41), suggesting that testosterone plays a key role in modulating HFD preference across sexes.

Despite the androgen-dependent increase in HFD preference and total energy intake, body weight gain did not differ between intact and orchiectomized males. Although absolute body weight before dietary choice was lower in orchiectomized males one week after surgery (**Table 2**), androgen deficiency did not affect subsequent weight gain during the dietary choice period (**Figure 3F**). These findings suggest that the increase in energy intake induced by androgens was insufficient to induce a greater body weight gain in this young adult model. The mechanism underlying this dissociation between energy intake and body weight gain remains unclear because energy expenditure was not measured. However, given that androgen deficiency did not affect energy intake or body weight gain under the ND-fed condition, it is possible that androgen deficiency preferentially reduces energy expenditure during HFD feeding through direct metabolic effects and/or reduced muscle mass (42, 43).

In contrast, in females, free-choice access to HFD did not affect energy intake in intact, E2(+), or E2(–) rats, nor did it affect body weight gain in intact or E2(+) rats, whereas body weight gain increased in E2(–) rats, in a manner similar to that observed in gonadally intact males (**Figure 3I**). Although energy expenditure was not measured in the present study, estrogens are known to enhance metabolic rate and physical activity (44–46). These results suggest that estrogens exert anorexigenic and anti-obesity effects even under free-choice conditions. Notably, unlike androgens, estrogens did not enhance total energy intake or HFD preference during HFD feeding, suggesting that their protective role is primarily mediated through homeostatic and metabolic regulation. These findings suggest that the regulation of the hedonic feeding response to an HFD may differ between sexes, rather than reflecting a reduced responsiveness to palatable food in females. However, the absence of an effect on HFD preference should be interpreted cautiously given the specific experimental conditions and relatively small sample size. In addition, female rats generally undergo pubertal maturation earlier than males at 8 weeks of age (47, 48), which may influence the maturation of food preference. Future studies using models that account for dynamic hormonal fluctuations will be important for clarifying these sex-specific mechanisms.

Previous studies have shown that female rats exhibit a greater preference for sweet tastes (49), and our previous study demonstrated that estradiol replacement enhances the sweet taste preference in ovariectomized rats (24). Together with the present findings, these results suggest that sex steroid hormones do not uniformly enhance preference for palatable foods (10–12). Rather, their effects on hedonic feeding appear to be stimulus-specific, and depending on the sensory and nutritional properties, such as sweetness or fat content. This selective modulation likely contributes to sex differences in dietary choice and energy intake regulation.

We evaluated ΔFosB expression in the NAc, a brain region crucial for hedonic feeding regulation, to assess its potential involvement in neural response to chronic HFD intake. ΔFosB is a marker of chronic neuronal activation that accumulates in response to repeated exposure to rewarding stimuli (35, 36). ΔFosB expression in the NAc was significantly greater in the M-HFD than in the M-ND group (**Figures 5A, B, D, E**) and tended to be higher in the Sham-HFD than in the Sham-ND group (**Figure 5H**). In addition, cumulative total energy intake was positively correlated with ΔFosB expression in the NAc core + shell in males, but not in females (**Supplementary Figures 2B, C**). Given that androgens increased both total energy intake and HFD intake under the HFD-fed condition, these results suggest that the androgen-induced increase in energy intake under HFD conditions is likely to be associated with the elevated ΔFosB expression in the NAc. However, our results do not specify the exact relationship between hedonic HFD intake and ΔFosB expression in the NAc. Other factors related to HFD feeding—including indirect influences such as motivation for dietary choice, metabolic status, or downstream neural adaptations—may contribute to the androgen-induced increase in ΔFosB expression during free-choice HFD exposure. Taken together, our current results merely suggest a potential link between androgen-induced HFD preference and ΔFosB expression. In females, exogenous estradiol increased ΔFosB expression compared to the F group, which maintained endogenous estrogen secretion (**Figures 5C, F**). However, we did not observe any effect of HFD feeding on ΔFosB expression in females (**Figures 5C, F, I**). These results suggest that relatively high levels of estradiol may upregulate chronic neural activity in the NAc, regardless of HFD feeding.

The specific site at which androgens exert their effects remains unclear. Androgen receptors (AR) are sparsely expressed in the NAc of the mouse brain (50). Therefore, it is possible that the AR-expressing neurons in other brain regions that project directly or indirectly to the NAc, such as the arcuate nucleus of the hypothalamus (51, 52), may indirectly facilitate the chronic neuronal activation in the NAc.

Maric et al. (29) reported results consistent with our study, showing that HFD feeding with a dietary choice increased total energy intake and body weight gain more significantly in male rats than in female rats. However, a discrepancy exists regarding HFD preference; they reported that the percentage of energy intake from the HFD was greater in females, whereas we observed a markedly higher preference in males. This discrepancy may be attributed to several methodological differences. First, the age at which HFD feeding commenced differed between the two studies: we initiated feeding at 8 weeks of age, whereas they began at 10 weeks. Furthermore, differences in feeding procedures and HFD composition likely influenced the outcomes. Notably, the HFD preference reported in their study for both sexes were lower than those observed in our study. Subtle variations in the HFD composition, such as lard and sucrose content, may have further contributed to the observed differences in HFD preference and intake between the two studies. Furthermore, dietary choice is strongly influenced by dietary factors and perceived palatability of foods. In the present study, we ensured the maintenance of diet palatability through daily replacement with fresh food, a procedure that may have preserved different sensory cues compared to studies with different feeding protocols. Given that dietary choice is highly sensitive to palatability, elucidating sex differences in food avoidance related to diet quality warrants further investigation.

While sex differences in HFD preference remain under debate, our findings provide a critical insight: endogenous androgens in males actively promote a preference for high-fat content during the early post-gonadal maturation period.

### 4.1 Limitations

The relatively small sample sizes (n = 6–9 per group) may have limited the statistical power to detect subtle effects, potentially increasing the risk of false negatives. However, our dataset demonstrated a clear alignment between effect sizes and P-values; differences in body weight and ingestive behavior exhibited large effect sizes with robust significance, confirming the reliability of our behavioral findings. In contrast, the ΔFosB data showed relatively smaller effect sizes and more marginal P-values, suggesting that we may have been unable to capture highly subtle or localized molecular changes. Future studies with larger sample sizes are warranted to fully validate and extend these underlying neurochemical mechanisms.

Androgens can be converted into estrogens by aromatase, an estrogen-synthesizing enzyme. A limitation of the present study is that we did not evaluate whether endogenous androgens exert their effects through AR or estrogen receptors (ERs). A potential solution to this issue would be to administer dihydrotestosterone (DHT), which acts exclusively on AR, to ORX rats and assess its effects. Further investigations are needed to clarify the effects of androgens on the HFD preference in male rats.

Another limitation of this study is that feeding behavior was not analyzed in relation to the estrous cycle, although feeding behavior has been shown to vary across the estrous cycle (9). Furthermore, the continuous estradiol replacement regimen used here does not mimic physiological cyclic fluctuations. Nevertheless, no clear estrous cycle-dependent variations in food intake were observed in intact female rats (data not shown). Although serum estradiol concentrations after the 30-day experiment were higher in the E2(+) group than in intact females, and considering the measurement of estradiol with ELISA may have limitation in reliability (53), energy intake and body weight gain in the E2(+) group were suppressed to levels comparable to those of intact controls. Asarian and Geary (9) reported that estrogens exert both phasic (cycle-dependent) and tonic (continuous) inhibitory effects on feeding. Therefore, the present findings provide meaningful insights into the overall tonic influence of estrogens on feeding regulation.

### 4.2 Perspectives

The findings of the present study highlight the importance of considering sex as a biological variable in the neuroendocrine regulation of feeding behavior. While estrogens play a protective role in regulating total energy intake and body weight, androgens enhance HFD preference, which may be mediated by modulation of hedonic feeding pathways, as suggested by ΔFosB expression in the NAc. These distinct mechanisms suggest that obesity prevention strategies should move beyond uniform approaches to address sex-specific hormonal drivers of overeating. Future studies aimed at evaluating motivational behaviors, identifying the precise neural circuits linking androgen signaling to these motivational pathways, and better modeling human food environments, will be essential for developing more effective and personalized clinical interventions.

## 5 Conclusions

The present study demonstrates that young adult males exhibit a greater preference for HFD than young adult females, with dietary choice. This sex difference appears to be mediated by androgens, which enhance HFD preference and total energy intake in HFD-fed males. In contrast, estrogens suppress weight gain and energy intake regardless of dietary conditions without significantly altering HFD preference in females. These findings indicate that androgens and estrogens divergently regulate HFD preference and total energy intake in young adult rats.

## Data Availability

The raw data supporting the conclusions of this article will be made available by the authors, without undue reservation.
