# Supplementary material for: Sex differences and the roles of sex steroids in energy intake regulation and high-fat diet preference during free-choice feeding in young adult rats
Source: Front Endocrinol (Lausanne). 2026 Jun 18;17:1864440. doi: 10.3389/fendo.2026.1864440 (PMC13322912; doi:10.3389/fendo.2026.1864440)
Supplement: Supplementary file 1 [file DataSheet1.docx]

Supplementary Material

# Supplementary Figures and Table

**Supplementary Figure 1.**

Representative images of ∆FosB-immunoreactive (∆FosB-ir) cells in the nucleus accumbens (NAc) related to Figure 5 (scale bar = 200 µm).

#

**Supplementary Figure 2.**

# Correlations between the density of ∆FosB immunoreactive (∆FosB-ir) cells in the nucleus accumbens (NAc) core + shell and cumulative total energy intake (A, B, C), cumulative high-fat diet (HFD) intake (D, E, F), and HFD preference (G, H, I) during the 20-day feeding period in Experiment 2. All correlations were analyzed by linear regression model. P values of covariates, factors, and their interaction were shown in the graph.

**Supplementary Table 1.**

Results from One- or Two-way ANOVA and unpaired Student’s t-test in Figures 1-5.

| **Figure** | | | **Analysis** | **Result** |  |  |  |  |  |  |  |  |
| --- | --- | --- | --- | --- | --- | --- | --- | --- | --- | --- | --- | --- |
| 1 | C | ∆B.W. | Two-way ANOVA |  |  | **Sum of Squares** | **df** | **Mean Square** | **F** | **p** | **η²** | **η²p** |
|  |  |  |  | Sex |  | 19757.1 | 1 | 19757.1 | 194.797 | <.001 | 0.892 | 0.907 |
|  |  |  |  | Diet |  | 333 | 1 | 333 | 3.283 | 0.085 | 0.015 | 0.141 |
|  |  |  |  | Interaction |  | 40.6 | 1 | 40.6 | 0.4 | 0.534 | 0.002 | 0.02 |
|  |  |  |  | Residuals |  | 2028.5 | 20 | 101.4 |  |  |  |  |
|  | E | Cum. energy intake | Two-way ANOVA |  |  | **Sum of Squares** | **df** | **Mean Square** | **F** | **p** | **η²** | **η²p** |
|  |  |  |  | Sex |  | 2180000 | 1 | 2180000 | 174.561 | <.001 | 0.893 | 0.897 |
|  |  |  |  | Diet |  | 6018 | 1 | 6018 | 0.481 | 0.496 | 0.002 | 0.023 |
|  |  |  |  | Interaction |  | 5160 | 1 | 5160 | 0.412 | 0.528 | 0.002 | 0.02 |
|  |  |  |  | Residuals |  | 250304 | 20 | 12515 |  |  |  |  |
| 2 | A | Serum [T] | Unpaired Student's T-Test |  | **Statistic** |  | **df** |  |  | **p** |  |  |
|  |  |  |  |  | -24.45 |  | 22 |  |  | <.001 |  |  |
|  | B | Serum [E2] | One-way ANOVA |  |  | **Sum of Squares** | **df** | **Mean Square** | **F** | **p** |  |  |
|  |  |  |  | Hormonal status |  | 2.51 | 2 | 1.2567 | 26.2 | <.001 |  |  |
|  |  |  |  | Residuals |  | 1.49 | 31 | 0.048 |  |  |  |  |
| 3 | C | ∆B.W. | Two-way ANOVA |  |  | **Sum of Squares** | **df** | **Mean Square** | **F** | **p** | **η²** | **η²p** |
|  |  |  |  | Sex |  | 8386 | 1 | 8386.1 | 183.35 | <.001 | 0.76 | 0.859 |
|  |  |  |  | Diet |  | 858 | 1 | 858.3 | 18.76 | <.001 | 0.078 | 0.385 |
|  |  |  |  | Interaction |  | 418 | 1 | 417.6 | 9.13 | 0.005 | 0.038 | 0.233 |
|  |  |  |  | Residuals |  | 1372 | 30 | 45.7 |  |  |  |  |
|  | F | ∆B.W. | Two-way ANOVA |  |  | **Sum of Squares** | **df** | **Mean Square** | **F** | **p** | **η²** | **η²p** |
|  |  |  |  | Hormonal status |  | 24 | 2 | 12 | 0.168 | 0.846 | 0.004 | 0.008 |
|  |  |  |  | Diet |  | 2328.3 | 1 | 2328.3 | 32.586 | <.001 | 0.43 | 0.437 |
|  |  |  |  | Interaction |  | 61.6 | 2 | 30.8 | 0.431 | 0.653 | 0.011 | 0.02 |
|  |  |  |  | Residuals |  | 3000.9 | 42 | 71.4 |  |  |  |  |
|  | I | ∆B.W. | Two-way ANOVA |  |  | **Sum of Squares** | **df** | **Mean Square** | **F** | **p** | **η²** | **η²p** |
|  |  |  |  | Hormonal status |  | 6064 | 2 | 3032.2 | 78.37 | <.001 | 0.722 | 0.781 |
|  |  |  |  | Diet |  | 504 | 1 | 503.5 | 13.01 | <.001 | 0.06 | 0.228 |
|  |  |  |  | Interaction |  | 124 | 2 | 62.1 | 1.6 | 0.213 | 0.015 | 0.068 |
|  |  |  |  | Residuals |  | 1702 | 44 | 38.7 |  |  |  |  |
| 4 | D | Cum. energy intake | Two-way ANOVA |  |  | **Sum of Squares** | **df** | **Mean Square** | **F** | **p** | **η²** | **η²p** |
|  |  |  |  | Sex |  | 1350000 | 1 | 1350000 | 238.4 | <.001 | 0.816 | 0.888 |
|  |  |  |  | Diet |  | 63941 | 1 | 63941 | 11.3 | 0.002 | 0.039 | 0.273 |
|  |  |  |  | Interaction |  | 71353 | 1 | 71353 | 12.6 | 0.001 | 0.043 | 0.295 |
|  |  |  |  | Residuals |  | 170457 | 30 | 5682 |  |  |  |  |
|  | E | Cum. energy intake | Two-way ANOVA |  |  | **Sum of Squares** | **df** | **Mean Square** | **F** | **p** | **η²** | **η²p** |
|  |  |  |  | Hormonal status |  | 160437 | 2 | 80219 | 12.04 | <.001 | 0.274 | 0.364 |
|  |  |  |  | Diet |  | 87638 | 1 | 87638 | 13.16 | <.001 | 0.15 | 0.239 |
|  |  |  |  | Interaction |  | 57842 | 2 | 28921 | 4.34 | 0.019 | 0.099 | 0.171 |
|  |  |  |  | Residuals |  | 279736 | 42 | 6660 |  |  |  |  |
|  | F | Cum. energy intake | Two-way ANOVA |  |  | **Sum of Squares** | **df** | **Mean Square** | **F** | **p** | **η²** | **η²p** |
|  |  |  |  | Hormonal status |  | 563913 | 2 | 281957 | 39.97 | <.001 | 0.597 | 0.645 |
|  |  |  |  | Diet |  | 43672 | 1 | 43672 | 6.19 | 0.017 | 0.046 | 0.123 |
|  |  |  |  | Interaction |  | 26817 | 2 | 13409 | 1.9 | 0.162 | 0.028 | 0.08 |
|  |  |  |  | Residuals |  | 310411 | 44 | 7055 |  |  |  |  |
|  | G | ND intake | Unpaired Student's T-Test |  | **Statistic** |  | **df** |  |  | **p** |  |  |
|  |  |  |  |  | -3.45 |  | 15 |  |  | 0.004 |  |  |
|  |  | HFD intake | Unpaired Student's T-Test |  | **Statistic** |  | **df** |  |  | **p** |  |  |
|  |  |  |  |  | 11.2 |  | 15 |  |  | <.001 |  |  |
|  | H | ND intake | One-way ANOVA |  |  | **Sum of Squares** | **df** | **Mean Square** | **F** | **p** | **η²** | **η²p** |
|  |  |  |  | Hormonal status |  | 28596 | 2 | 14298 | 3.56 | 0.047 | 0.253 | 0.253 |
|  |  |  |  | Residuals |  | 84401 | 21 | 4019 |  |  |  |  |
|  |  | HFD intake | One-way ANOVA |  |  | **Sum of Squares** | **df** | **Mean Square** | **F** | **p** | **η²** | **η²p** |
|  |  |  |  | Hormonal status |  | 337928 | 2 | 168964 | 13.2 | <.001 | 0.556 | 0.556 |
|  |  |  |  | Residuals |  | 269810 | 21 | 12848 |  |  |  |  |
|  | I | ND intake | One-way ANOVA |  |  | **Sum of Squares** | **df** | **Mean Square** | **F** | **p** | **η²** | **η²p** |
|  |  |  |  | Hormonal status |  | 41066 | 2 | 20533 | 2.71 | 0.089 | 0.198 | 0.198 |
|  |  |  |  | Residuals |  | 166659 | 22 | 7575 |  |  |  |  |
|  |  | HFD intake | One-way ANOVA |  |  | **Sum of Squares** | **df** | **Mean Square** | **F** | **p** | **η²** | **η²p** |
|  |  |  |  | Hormonal status |  | 264589 | 2 | 132294 | 6.58 | 0.006 | 0.374 | 0.374 |
|  |  |  |  | Residuals |  | 442294 | 22 | 20104 |  |  |  |  |
|  | M | HFD preference | Unpaired Student's T-Test |  | **Statistic** |  | **df** |  |  | **p** |  |  |
|  |  |  |  |  | 4.71 |  | 15 |  |  | <.001 |  |  |
|  | N | HFD preference | One-way ANOVA |  |  | **Sum of Squares** | **df** | **Mean Square** | **F** | **p** | **η²** | **η²p** |
|  |  |  |  | Hormonal status |  | 165 | 2 | 82.3 | 4.74 | 0.02 | 0.311 | 0.311 |
|  |  |  |  | Residuals |  | 364 | 21 | 17.4 |  |  |  |  |
|  | O | HFD preference | One-way ANOVA |  |  | **Sum of Squares** | **df** | **Mean Square** | **F** | **p** | **η²** | **η²p** |
|  |  |  |  | Hormonal status |  | 141 | 2 | 70.4 | 1.45 | 0.256 | 0.117 | 0.117 |
|  |  |  |  | Residuals |  | 1067 | 22 | 48.5 |  |  |  |  |
| 5 | A | ∆FosB NAc core+shell | Two-way ANOVA |  |  | **Sum of Squares** | **df** | **Mean Square** | **F** | **p** | **η²** | **η²p** |
|  |  |  |  | Sex |  | 183 | 1 | 183 | 0.451 | 0.507 | 0.011 | 0.016 |
|  |  |  |  | Diet |  | 2539 | 1 | 2539 | 6.255 | 0.019 | 0.158 | 0.188 |
|  |  |  |  | Interaction |  | 2340 | 1 | 2340 | 5.766 | 0.023 | 0.146 | 0.176 |
|  |  |  |  | Residuals |  | 10958 | 27 | 406 |  |  |  |  |
|  | B | ∆FosB NAc core+shell | Two-way ANOVA |  |  | **Sum of Squares** | **df** | **Mean Square** | **F** | **p** | **η²** | **η²p** |
|  |  |  |  | Hormonal status |  | 341 | 2 | 171 | 0.355 | 0.704 | 0.013 | 0.018 |
|  |  |  |  | Diet |  | 4177 | 1 | 4177 | 8.674 | 0.005 | 0.162 | 0.182 |
|  |  |  |  | Interaction |  | 2497 | 2 | 1249 | 2.593 | 0.088 | 0.097 | 0.117 |
|  |  |  |  | Residuals |  | 18779 | 39 | 482 |  |  |  |  |
|  | C | ∆FosB NAc core+shell | Two-way ANOVA |  |  | **Sum of Squares** | **df** | **Mean Square** | **F** | **p** | **η²** | **η²p** |
|  |  |  |  | Hormonal status |  | 6158 | 2 | 3079 | 6.417 | 0.004 | 0.222 | 0.23 |
|  |  |  |  | Diet |  | 665 | 1 | 665 | 1.386 | 0.246 | 0.024 | 0.031 |
|  |  |  |  | Interaction |  | 289 | 2 | 144 | 0.301 | 0.742 | 0.01 | 0.014 |
|  |  |  |  | Residuals |  | 20629 | 43 | 480 |  |  |  |  |
|  | D | ∆FosB NAc core | Two-way ANOVA |  |  | **Sum of Squares** | **df** | **Mean Square** | **F** | **p** | **η²** | **η²p** |
|  |  |  |  | Sex |  | 310 | 1 | 310 | 1.16 | 0.29 | 0.028 | 0.041 |
|  |  |  |  | Diet |  | 1477 | 1 | 1477 | 5.54 | 0.026 | 0.132 | 0.17 |
|  |  |  |  | Interaction |  | 2180 | 1 | 2180 | 8.18 | 0.008 | 0.195 | 0.232 |
|  |  |  |  | Residuals |  | 7198 | 27 | 267 |  |  |  |  |
|  | E | ∆FosB NAc core | Two-way ANOVA |  |  | **Sum of Squares** | **df** | **Mean Square** | **F** | **p** | **η²** | **η²p** |
|  |  |  |  | Hormonal status |  | 273 | 2 | 137 | 0.377 | 0.689 | 0.014 | 0.019 |
|  |  |  |  | Diet |  | 2137 | 1 | 2137 | 5.888 | 0.02 | 0.113 | 0.131 |
|  |  |  |  | Interaction |  | 2296 | 2 | 1148 | 3.163 | 0.053 | 0.122 | 0.14 |
|  |  |  |  | Residuals |  | 14154 | 39 | 363 |  |  |  |  |

|  | F | ∆FosB NAc core | Two-way ANOVA |  |  | **Sum of Squares** | **df** | **Mean Square** | **F** | **p** | **η²** | **η²p** |
| --- | --- | --- | --- | --- | --- | --- | --- | --- | --- | --- | --- | --- |
|  |  |  |  | Hormonal status |  | 5215 | 2 | 2607 | 8.627 | <.001 | 0.275 | 0.286 |
|  |  |  |  | Diet |  | 336 | 1 | 336 | 1.113 | 0.297 | 0.018 | 0.025 |
|  |  |  |  | Interaction |  | 427 | 2 | 214 | 0.707 | 0.499 | 0.023 | 0.032 |
|  |  |  |  | Residuals |  | 12995 | 43 | 302 |  |  |  |  |
|  | G | ∆FosB NAc shell | Two-way ANOVA |  |  | **Sum of Squares** | **df** | **Mean Square** | **F** | **p** | **η²** | **η²p** |
|  |  |  |  | Sex |  | 16.68 | 1 | 16.68 | 0.4445 | 0.511 | 0.014 | 0.016 |
|  |  |  |  | Diet |  | 142.59 | 1 | 142.59 | 3.7995 | 0.062 | 0.121 | 0.123 |
|  |  |  |  | Interaction |  | 2.87 | 1 | 2.87 | 0.0763 | 0.784 | 0.002 | 0.003 |
|  |  |  |  | Residuals |  | 1013.24 | 27 | 37.53 |  |  |  |  |
|  | H | ∆FosB NAc shell | Two-way ANOVA |  |  | **Sum of Squares** | **df** | **Mean Square** | **F** | **p** | **η²** | **η²p** |
|  |  |  |  | Hormonal status |  | 10.8 | 2 | 5.41 | 0.215 | 0.808 | 0.008 | 0.011 |
|  |  |  |  | Diet |  | 338.4 | 1 | 338.35 | 13.438 | <.001 | 0.249 | 0.256 |
|  |  |  |  | Interaction |  | 28 | 2 | 13.98 | 0.555 | 0.578 | 0.021 | 0.028 |
|  |  |  |  | Residuals |  | 982 | 39 | 25.18 |  |  |  |  |
|  | I | ∆FosB NAc shell | Two-way ANOVA |  |  | **Sum of Squares** | **df** | **Mean Square** | **F** | **p** | **η²** | **η²p** |
|  |  |  |  | Hormonal status |  | 137.6 | 2 | 68.81 | 1.58 | 0.218 | 0.066 | 0.068 |
|  |  |  |  | Diet |  | 55.3 | 1 | 55.34 | 1.271 | 0.266 | 0.027 | 0.029 |
|  |  |  |  | Interaction |  | 16.9 | 2 | 8.46 | 0.194 | 0.824 | 0.008 | 0.009 |
|  |  |  |  | Residuals |  | 1872.2 | 43 | 43.54 |  |  |  |  |
